# Opening up new horizons for psychiatric genetics in the Russian Federation: moving toward a national consortium

**DOI:** 10.1038/s41380-019-0354-z

**Published:** 2019-01-21

**Authors:** Olga Yu. Fedorenko, Vera E. Golimbet, Svetlana А. Ivanova, Аnastasia Levchenko, Raul R. Gainetdinov, Arkady V. Semke, German G. Simutkin, Аnna E. Gareeva, Аndrey S. Glotov, Anna Gryaznova, Ivan Y. Iourov, Evgeny M. Krupitsky, Igor N. Lebedev, Galina E. Mazo, Vasily G. Kaleda, Lilia I. Abramova, Igor V. Oleichik, Yulia A. Nasykhova, Regina F. Nasyrova, Anton E. Nikolishin, Evgeny D. Kasyanov, Grigory V. Rukavishnikov, Ilgiz F. Timerbulatov, Vadim M. Brodyansky, Svetlana G. Vorsanova, Yury B. Yurov, Tatyana V. Zhilyaeva, Anzhelika V. Sergeeva, Elena A. Blokhina, Edwin E. Zvartau, Anna S. Blagonravova, Lyubomir I. Aftanas, Nikolay А. Bokhan, Zurab I. Kekelidze, Tatyana V. Klimenko, Irina P. Anokhina, Elza K. Khusnutdinova, Tatyana P. Klyushnik, Nikolay G. Neznanov, Vadim A. Stepanov, Thomas G. Schulze, Аleksandr О. Kibitov

**Affiliations:** 1 Mental Health Research Institute, Tomsk National Research Medical Center of Russian Academy of Sciences, Tomsk, Russian Federation; 2 National Research Tomsk Polytechnic University, Tomsk, Russian Federation; 3 Mental Health Research Center, Moscow, Russian Federation; 4 Institute of Translational Biomedicine, Saint Petersburg State University, Saint Petersburg, Russian Federation; 5 Institute of Biochemistry and Genetics, Ufa Federal Research Center, Russian Academy of Sciences, Ufa, Russian Federation; 6 Federal State Educational Institution of Highest Education Bashkir State Medical University of Public Health Ministry of Russian Federation, Ufa, Russian Federation; 7 Laboratory of Biobanking and Genomic Medicine of Institute of Translational Biomedicine, Saint Petersburg State University, Saint Petersburg, Russian Federation; 8 Institute of Psychiatric Phenomics and Genomics (IPPG), University Hospital, LMU, Munich, Germany; 9 V.M. Bekhterev National Medical Research Center for Psychiatry and Neurology, Saint Petersburg, Russian Federation; 10 Research Institute of Medical Genetics, Tomsk National Research Medical Center of Russian Academy of Sciences, Tomsk, Russian Federation; 11 Serbsky National Medical Research Center on Psychiatry and Addictions, Moscow, Russian Federation; 12 Veltischev Research and Clinical Institute for Pediatrics, the Pirogov Russian National Research Medical University, Moscow, Russian Federation; 13 Privolzhskiy Research Medical University, Nizhny Novgorod, Russian Federation; 14 First Saint Petersburg Pavlov State Medical University, Saint Petersburg, Russian Federation; 15 Federal State Scientific Budgetary Institution “Scientific Research Institute of Physiology and Basic Medicine,”, Novosibirsk, Russian Federation; 16 National Research Tomsk State University, Tomsk, Russian Federation

**Keywords:** Schizophrenia, Depression, Addiction, Diagnostic markers

## Abstract

We provide an overview of the recent achievements in psychiatric genetics research in the Russian Federation and present genotype-phenotype, population, epigenetic, cytogenetic, functional, ENIGMA, and pharmacogenetic studies, with an emphasis on genome-wide association studies. The genetic backgrounds of mental illnesses in the polyethnic and multicultural population of the Russian Federation are still understudied. Furthermore, genetic, genomic, and pharmacogenetic data from the Russian Federation are not adequately represented in the international scientific literature, are currently not available for meta-analyses and have never been compared with data from other populations. Most of these problems cannot be solved by individual centers working in isolation but warrant a truly collaborative effort that brings together all the major psychiatric genetic research centers in the Russian Federation in a national consortium. For this reason, we have established the Russian National Consortium for Psychiatric Genetics (RNCPG) with the aim to strengthen the power and rigor of psychiatric genetics research in the Russian Federation and enhance the international compatibility of this research.

The consortium is set up as an open organization that will facilitate collaborations on complex biomedical research projects in human mental health in the Russian Federation and abroad. These projects will include genotyping, sequencing, transcriptome and epigenome analysis, metabolomics, and a wide array of other state-of-the-art analyses. Here, we discuss the challenges we face and the approaches we will take to unlock the huge potential that the Russian Federation holds for the worldwide psychiatric genetics community.

## Introduction

About 15 years ago, the Russian geneticist E. Rogaev published a paper on academic psychiatry in Russia [1]. However, this interesting report did not provide sufficient details on the direction of major psychiatric genetics research projects being performed at the leading psychiatric centers in Russia.

The problems of genetic research in psychiatry are well known, i.e., the weak effects of multiple genes with an additive and interactive effect; the ambiguity of hypotheses about pathogenesis; psychiatric and somatic comorbidity; the lack of reliable and verified laboratory tests; ethnic stratification; and blurred and overlapping diagnostic categories. Some of these problems are particularly relevant to research in the Russian Federation. The population of the Russian Federation, about 144 million people, is very unevenly distributed over a large area and is polyethnic (it includes more than 160 ethnic groups of East and North European and North and Central Asian descent). The predominant ethnic group is Russian (~81%). All people in the Russian Federation belong to one of nine language families: Indo-European, Kartvelian, Ural-Yukagir, Altaic, Eskimo-Aleutian, North Caucasian, Yenisei, Sino-Tibetan, and Chukchi-Kamchatka. As a result of significant migration and active interethnic mixing, the population is a complex ethnic conglomerate.

The genetic backgrounds of mental illnesses in the polyethnic and multicultural population of the Russian Federation are still understudied. Genetic, genomic, and pharmacogenetic data from the Russian Federation are not adequately represented in the international scientific literature and thus are not available for meta-analyses and have never been compared with data from other populations.

Most of these problems cannot be solved by individual centers working in isolation but warrant a truly collaborative effort that brings together all the psychiatric genetic research centers in the Russian Federation in a national consortium. Such a consortium will strengthen the power and rigor of psychiatric genetics research in the Russian Federation and enhance the international compatibility of this research.

This paper provides an update on the progress of psychiatric genetics research at centers in the Russian Federation and the international collaborations of these centers and describes the framework and goals of the recently founded Russian National Consortium for Psychiatric Genetics (RNCPG).

## The modern landscape of psychiatric genetics in the Russian Federation

Currently, there are eight psychiatric genetic research centers in five cities in the Russian Federation: the Mental Health Research Center (MHRC) and the Serbsky National Medical Research Center on Psychiatry and Addictions (SNMRCPA) in Moscow; the Mental Health Research Institute (MHRI) and the Research Institute of Medical Genetics (RIMG), Tomsk National Research Medical Center in Tomsk; V.M. Bekhterev National Medical Research Center for Psychiatry and Neurology (BNMRCPN) and the Institute of Translational Biomedicine (ITBM), Saint Petersburg State University (SPBU) in St. Petersburg; the Institute of Biochemistry and Genetics (IBG), Ufa Scientific Center in Ufa; and Privolzhskiy Research Medical University in Nizhny Novgorod (PRMU).^1^ Below, we present some of the past and current research in psychiatric genetics at these centers.

The Clinical Genetics Laboratory (MHRC, Moscow) has been working in the field of psychiatric genetics and psychiatric genomics for the last three decades. The main focus of its research is molecular genetic studies of schizophrenia (SZ) and affective disorders. Several thousands of clinically well-characterized DNA samples have been collected and used in different kinds of studies, i.e., case-control and family-based association studies and studies of endophenotypes and gene-environment interactions.

Researchers at the MHRC collaborate with other centers for psychiatric genetics worldwide, including the Psychiatric Genomics Consortium (PGC). In addition to 4500 patients from 11 European locations, the first large GWAS in SZ included 500 patients from the Russian population [2]. Later, samples from Russian patients with SZ and bipolar disorder (BD) were used in several collaborative projects, including some with the PGC. The results of these studies contributed to a better understanding of and provided new insights into the genetic architecture of these disorders [3–6]. Also, in 2013 the IBG (Ufa) collaborated with the PGC to perform a GWAS of individuals with SZ who were of Russian, Tatar, or Bashkir descent; the aim was to search for ethnically specific genetic risk markers for SZ.

In 2010, the RIMG (Tomsk) joined the European Union funded (FP7-HEALTH) ADAMS project, which searched for genomic variations underlying Alzheimer’s disease (AD), alcoholism, SZ, and cognition traits. Within ADAMS, the RIMG performed a population genomic study of genetic markers associated with neurological and mental diseases (AD, Parkinson’s disease [PD], SZ, and cognitive performance); the study demonstrated considerable between-population variability in allele frequencies across multiple Eurasian populations (eight populations were included, representing Eastern Europe, Central Asia, Northern Asia, and Siberia) [7]. Population differentiation measured by Wright’s F statistic (Fst), which corresponds to the proportion of between-population variability in the total genetic variance, varied widely across the loci studied (from 0.006 to 0.146), and the mean Fst for all 55 single nucleotide polymorphisms (SNPs) was 0.045. The mean Fst for AD (0.029) was substantially lower than for PD (0.044), SZ (0.054), and cognitive performance (0.049). The allele frequencies tended to correlate between populations according to their geographical locations. The data indicated that wide inter-population variation exists in the frequency of alleles associated with common diseases, even on a sub-continental level [7].

A microarray for genotyping ten SNPs associated with the sporadic form of AD was developed by the Engelhardt Institute of Molecular Biology in collaboration with the RIMG [8, 9].

In a replication study at the RIMG, 50 SNPs found in GWASs of SZ, AD, and cognitive endophenotypes were studied in Russian and Kazakh patients with AD and SZ [10–12]. SNPs in regions of eight genes, including *APOЕ*, were associated with AD in the Russian participants. The associations between 10 genetic markers and SZ were replicated in Russians, and the associations between 8 genetic markers and SZ were replicated in Kazakhs. Among the genetic markers associated with AD and SZ in the Russian population, 3 genes were common for both diseases, indicating an overlap of pathogenetic mechanisms between AD and SZ; this overlap is probably mediated by cognitive endophenotypes (manuscript in preparation).

The IBG performed a study in individuals with SZ from the Russian, Tatar, and Bashkir ethnic groups to investigate 88 polymorphisms of 23 candidate genes implicated in neurotransmission and neuronal development. The study found differences in *GRIN2B* [13], *NTRK3*, *NXPH1* [14], *GRIA2* [15], *GRIK2*, *RGS2* [16], and *TPH1* [17] between SZ patients from the Russian and Tatar ethnic groups. A further study identified risk markers for unipolar depression in *SLC6A4*, *HTR2A*, *TPH1*, *MAOA*, *NTRK2*, *NTRK3*, *NRXN1*, *NXPH1*, and *MTHFR* in both Russians and Tatars [18, 19].

A study performed at the MHRI (Tomsk) showed an association of (N251S)-PIP5K2A with SZ [20]. At PRMU (Nizhny Novgorod), an association study of single-carbon metabolism genes with SZ, the severity of negative and cognitive symptoms, the clinical course of the disease, and the side effects of antipsychotic therapy has been ongoing since 2013 [21, 22].

Genotype-phenotype studies at the Clinical Genetics Laboratory (MHRC) have focused on the clinical characteristics of SZ, in particular on cognitive deficits, a core and enduring feature of the illness. Associations between candidate genes and impairments in short-term and verbal memory and selective attention were identified in large groups of patients with SZ [23–25]. The laboratory also looked for genetic polymorphisms associated with some important but understudied features of social behavior and social cognition in SZ (Machiavellianism, facial emotion recognition, and theory of mind) [26, 27]. Currently, its efforts are concentrated on developing approaches to investigate the genetic background of cognitive reserve and resilience in SZ. Its research also focuses on the relationship between genes involved in inflammation and personality traits that predispose to SZ; the level of premorbid social functioning in SZ patients; the severity of the main clinical syndromes of SZ [28, 29]; and genes that exert a modifying effect on the course, clinical features, and outcome of SZ [30–32].

Research at the Department of Addictions at the BNMRCPN and the Laboratory of Clinical Psychopharmacology of Addictions, First Saint Petersburg Pavlov State Medical University, places great emphasis on identifying genetic variants involved in alcohol and opioid addictions [33–35]. Since 1999, when an association was found between dopamine genes and alcohol dependence in the Russian population [36], the molecular genetics laboratory at the SNMRCPA (Moscow) has focused on genetic risk markers and polygenic risk scores for opioid and alcohol dependence, including severe complications of alcohol withdrawal syndrome, and on the search for genetic markers of possible “hereditary” forms of the disorders and the associated courses, clinical features, and outcomes [37–39].

An RIMG (Tomsk) project funded by the Russian Science Foundation (RSF) aimed to elucidate the link between the genetic components of neuropsychiatric diseases and normal variability in cognitive functions in the elderly. Some alleles and haplotypes predisposing to AD were found to be associated with a worse cognitive status in elderly people without a diagnosis of AD [40–42]. It is likely that the normal age-dependent variability in cognitive functions can be considered as an endophenotype of AD. Currently, the search for new genetic markers of the variability in cognitive functions is being conducted through exome-wide association studies.

Two sequencing studies at SPBU revealed a list of several ultra-rare or previously unreported variants in several brain-expressed genes in patients with SZ and schizotypy that were absent in healthy controls [43, 44]. Future functional studies will assess the pathogenic role of these variants, including one interesting candidate found to be damaging in silico [45].

Over 1500 children with idiopathic intellectual disability were examined as part of the European Union project “Improving Diagnoses of Mental Retardation in Children in Eastern Europe and Central Asia through Genetic Characterisation and Bioinformatics/Statistics” (CHERISH), and the RIMG identified several novel chromosomal microdeletions and microduplications [46–49]. All clinical and molecular protocols were introduced into the Genetics Clinic of the RIMG in Tomsk and are now available to patients.

In parallel, the Molecular Brain Genetics Laboratory at the MHRC evaluated variomes in children with intellectual disability and revealed a series of previously undescribed genomic and epigenomic abnormalities (i.e., runs of homozygosity affecting imprinted genomic loci) associated with this devastating condition [50, 51].

Since the late 1980s, the Laboratory of Cytogenetics and Genomics of Psychiatric Diseases and the Molecular Brain Genetics Laboratory at the MHRC (Moscow) have focused on studying gene and chromosomal mutations in psychiatric diseases, and in 1998 they entered the chromosome 18 workshop [52]. The laboratories had the original idea to look for brain-specific genetic defects, and they thereby uncovered mosaic chromosome abnormalities in the SZ brain [53], suggested brain-confined mosaicism as a mechanism of mental illness [54] and found somatic mosaicism to be common in autism [55]. Additionally, brain- and chromosome-specific instability (aneuploidy) was reported in SZ [56] and in neurodegenerative diseases (ataxia-telangiectasia and AD) [57]. Consequently, chromosome instability (a recognized genetic mechanism of cancer) was shown to underlie neurodegeneration [58]. As a result, the laboratories proposed the DNA replication stress hypothesis of AD, which links the amyloid and cell cycles (the abortive neuronal cell cycle is hypothesized to cause neurodegeneration) [59]. Further studies revealed a link between AD and aging or cell senescence that was related to chromosome instability [60]. The laboratories’ latest studies have shed some light on the genetic mechanisms of comorbidity in psychiatric disorders, which may be associated with similar patterns of brain-specific chromosome instability in at least some cases [61]. Recently, these pioneering efforts were recognized by the “Brain Somatic Mosaicism Network” (https://www.nimh.nih.gov/news/events/2017/brain-somatic-mosaicism-network-investigators-workshop.shtml). Finally, these laboratories identified a treatment for psychiatric diseases associated with chromosomal imbalances (previously incurable conditions) by uncovering pathways disrupted by a genomic pathology that can be corrected by diet and conventional medications [62].

The Clinical Genetics Laboratory at the MHRC (Moscow) is currently studying epigenetic markers associated with neurocognitive deficits in SZ by using the SMRT-BS method, which is based on the third-generation high-throughput sequencing platform PacBio. The first results showed variability in the methylation level of different regions within the promoter of the reelin gene [63].

To study the functional relevance of newly discovered genetic variants for SZ ex vivo, researchers at the MHRC have started to collect primary cell cultures derived from the olfactory epithelium of patients with SZ.

Researchers at the MHRI (Tomsk), in collaboration with the Institute of Physiology, University of Tuebingen (Germany), demonstrated the functional relevance of (N251S)-PIP5K2A in SZ via deranged regulation of neuronal KCNQ potassium channels and EAAT3 excitatory amino acid transporters [64, 65]. In collaboration with the Institute of Cytology and Genetics (Novosibirsk, Russia), the researchers at the RIMG (Tomsk) obtained induced pluripotent stem cells (IPSCs) from patients with 3p26.3 microdeletions and microduplications affecting *CNTN6*, whose role in the pathogenesis of intellectual disability had not been previously described. The IPSCs were further differentiated into cortical neurons, and the researchers found that the *CNTN6* expression level from the duplicated allele was significantly decreased, leading to haploinsufficiency [66]. This finding provides a novel molecular explanation for the origin of overlapping clinical and neurodevelopmental features in patients with reciprocal copy number variations.

The Enhancing Neuroimaging Genetics Through Meta Analysis (ENIGMA) network comprises over 30 working groups worldwide (http://enigma.ini.usc.edu/) and includes three groups from Russia that are working on major depressive disorder and SZ: (1) the MHRC (Moscow), (2) the MHRI (Tomsk), and (3) the Scientific Research Institute of Physiology and Basic Medicine (Novosibirsk). These Russian institutions provided MRI data and DNA from patients with major depressive disorder and SZ and from healthy individuals for the genetic association studies of brain abnormalities in these disorders [67, 68] and for the studies mapping cortical brain asymmetry in healthy individuals [69].

For the last decade, the Laboratory of Molecular Genetics and Biochemistry (MHRI, Tomsk) has been working in the field of pharmacogenetics of mental and neurodegenerative disorders. They work in collaboration with the Groningen Research Institute of Pharmacy, University of Groningen (Netherlands). The results of the studies in SZ are summarized in Table 1.

**Table 1:** Gene association findings of studies on antipsychotic-induced side effects in Russian inpatients with schizophrenia

Sample size, *N*	Patients with side effects, *n*	Gene	Associated allele(s) and genotype(s)	Ref.
146	43	*DRD3*	Ser9Gly association with TDlt	[82]
		*HTR2C*	Cys23Ser association with TDlt	
146	43	*MnSOD*	Ala-9Val association with TDof	[83]
401	127	*GRIN2A*	rs1345423 C allele association with TD and TDof; rs11646587 A allele association with TDof; rs7206256 А allele association with TDof; rs7190619 AG genotype association with TDof	[84–86]
		*GRIN2B*	rs2192970 association with TD	
146	43	*CYP17A1*	Cyp17 (T34C, rs743572) TC or TT genotypes association with TD	[87]
319	206	*CYP1A2*	CYP1A2*1F (-163C>A, rs762551) association with TD	[88]
		*CYP2D6*	CY2D6*4 (1846G>A) and genotype A/A association with TDlt	
465	131	*PIP5K2A*	rs10828317 (N251S) association with TD	[89]
146	43	*ADORA2A*	rs3032740 (2592C/Tins) no association with TD	[90]
401	127	*GRIN2A*	rs1345423 association with TD in both Dutch and Russian patient populations	[91]
446	227	*HTR2C*	X-chromosome haplotype (rs569959 and rs17326429) association with HPRL	[92]
128	66	*CYP2D6*	rs3892097 association with HPRL	[93]
		*HTR2C*	rs6318 association with HPRL	
443	227	*PRL*	rs1341239 (-1149 G/T) association with HPRL	[94]

*TD* tardive dyskinesia, *TDof* orofaciolingual tardive dyskinesia, *TDlt* limb-truncal tardive dyskinesia, *HPRL* hyperprolactinemia

The BNMRCPN (Saint Petersburg) performed a study in patients with acute psychosis treated with olanzapine or haloperidol and found no evidence that mRNA levels of the dopamine D4 receptor and serotonin 2A receptor in peripheral blood mononuclear cells are biomarkers of treatment response [70]. Another study in patients with SZ performed at the IBG (Ufa) showed that polymorphism in genes of the serotoninergic and dopaminergic systems affects individual sensitivity to haloperidol [71]. A study at the Molecular Genetics Laboratory of the SNMRCPA (Moscow) in a cohort of inpatients with SZ found that CYP2D6 metabolic activity affects the mean antipsychotic daily dose only in the presence of the *DRD2*/*ANKK1* Taq1A T allele, but CYP2D6 metabolic activity correlates with the duration of the hospital stay independent of *DRD2* Taq1A [72]. A subsequent study revealed that polymorphism of *SCL6A3* can affect the safety of haloperidol in patients with alcohol use disorder [73].

Two studies at the MHRI (Tomsk) found that the polymorphisms *AKT1* rs1130214 and *GSK3B* rs334558 are associated with antidepressant treatment response [74, 75].

A pharmacogenetic component of the collaborative double-blind, double-dummy, placebo-controlled randomized clinical trial of implantable naltrexone vs. oral naltrexone and placebo for opioid dependence (Molecular Genetics Laboratory of the SNMRCPA, Moscow; Department of Addictions, BNMRCPN; the Laboratory of Clinical Psychopharmacology of Addictions, First Saint Petersburg Pavlov State Medical University; and Baylor College of Medicine, TX, USA) showed the joint effect of opioid receptor genes and genes of the dopaminergic system on treatment outcomes [76]. In another study, this collaborative group showed the additive effect of opioid receptor genes and dopaminergic system genes on outcomes of treatment of opioid dependence with oral naltrexone and guanfacine [77].

## Opening up new horizons: the Russian National Consortium for Psychiatric Genetics (RNCPG)

As detailed above, a wide range of high-quality studies have been performed at individual research centers and in “center-to-center” collaborations in Russia, and various centers have participated in international projects. Nevertheless, it became clear that the psychiatric genetic research centers in Russia need to work together even more closely to improve and accelerate development in the field of psychiatric genetics in Russia. The tasks at hand cannot be solved by individual centers working in isolation but warrant a truly collaborative effort that brings together all the centers in a Russian-wide consortium.

The first scientific conference on psychiatric genetics in Russia, “Genetics and epigenetics of mental illnesses,” took place in Moscow in November 2017. It was organized by the MHRC and SNMRCPA (Moscow) and the Russian Society of Psychiatrists and supported by the Russian Foundation for Basic Research (RFBR). At this conference, the Russian National Consortium for Psychiatric Genetics (RNCPG) was established and all the primary members wrote the memorandum of understanding. The Consortium is set up as an open organization that will enable collaborations within the Russian Federation and worldwide (http://rncpg.org).

## Specific aims and goals of the RNCPG

The primary goal of the Consortium is to foster a better understanding of the genetic basis of mental illnesses with the aim to improve their prevention and treatment in the population of the Russian Federation.

To achieve this goal, the Consortium members have agreed to:

1. unite the scientific, clinical, and technological capabilities of the leading Russian research centers in the field of psychiatric genetics;
2. establish and maintain Russia’s largest collection of biomaterials from people with mental illnesses and population-matched controls. In the initial stage, i.e., by 2020, we expect to collect biomaterials from the following numbers of people: ~15,000 with SZ, ~10,000 with major depressive disorder, ~3000 with BD, ~8000 with alcohol and drug dependence, ~3000 with eating disorders, and ~20,000 healthy controls;
3. ensure that genetic and biomedical interdisciplinary research into the etiology, pathogenesis, and efficacy of pharmacotherapy of mental diseases in the population of Russia is performed according to modern scientific and technological standards;
4. facilitate the participation of Russian scientists and studies in the Russian population in international collaborations;
5. develop guidelines for clinical practice that apply the results of genetic studies in the Russian population.

Our current priorities in the Consortium are as follows:

1. Integrate genomic, pharmacogenomic, and epigenomic projects within the Consortium, with an emphasis on the importance of prospective studies; provide opportunities for biological research beyond genomics.
2. Integrate epidemiological studies of psychiatric disorders that compare Russians with ethnic groups from other countries; efficiently account for ethnic heterogeneity.
3. Enable the standardized assessment of a wide range of both traditional and novel clinical phenotypes; ensure the highest standards of scientific practice at all levels of psychiatric genetic research and facilitate true interdisciplinary research.

We are currently considering reorganizing the structure of the RNCPG to better correspond with our goals. We propose that the RNCPG should include six scientific research centers, each with their own psychiatric hospital; eight psychiatric hospitals that act as RNCPG partners; the SPBU Biobank, which is the technological hub of the RNCPG; and the- Institute of Translational Biomedicine in SPBU, the translational core of the RNCPG. All these units will need to work together in the RNCPG to generate high-quality genomic data that can be quickly translated into clinical practice and enable the development of treatment guidelines.

Today, the RNCPG comprises the following research centers: MHRC; SNMRCPA; MHRI; BNMRCPN; IBG; and PRMU. It represents a voluntary association of research scientists and physicians that allows psychiatrists and geneticists to collaborate closely from the initial planning of projects to data processing and final discussions. These features enable the RNCPG to construct high quality, correct phenotypes for a broad spectrum of diseases and disorders. As a result, in May 2018 the Consortium became a special section in the Russian Society of Psychiatrists.

No special diagnostic groups are planned, and every collaboration project inside the Consortium is flexible and open to members and associated participants.

Most of the centers in the Consortium have their own psychiatric hospital. The Consortium already collaborates with the leading psychiatric hospitals in the Russian Federation; these eight hospitals have more than 10,000 inpatient beds and together treat more than 12,000 outpatients per year. In addition, six hospitals from other regions of the country have agreed to collaborate with the Consortium.

The Consortium operates on the basis of the international principles of medical ethics, and all biosamples and participant data are used only after informed consent has been obtained.

All the biomaterials will be processed inside the Russian Federation according to the law, regulations, and policies related to human biomaterial. The fully coded and anonymized data sharing procedure will be included in specific protocols in every international collaboration project.

## SPBU Biobank as the technological hub for the RNCPG

Founded in 2015, the biobank of SPBU focuses on complex biomedical research projects on human health and longevity. Since the year it was founded, the biobank has been involved in the project “Genome of Russia,” which aims to analyze the whole-genome sequence of 2500 men and women from the various regions of Russia.

SPBU biobank joined the RNCPG when it was founded at the end of 2017 and began processing and providing long-term storage of blood and DNA samples for the Consortium. Before then, there was no biobank in Russia specializing in the standardized collection and storage of biological samples from patients with psychiatric diseases. Today, the SPBU biobank constitutes the Consortium’s technological core and hub for biosamples and serves as the central unit for storing and processing all the biomaterials collected at the member centers. Processing includes genotyping, sequencing, transcriptome and epigenome analysis, metabolomics, and a wide array of other state-of-the-art analyses. Like all biobanks, SPBU biobank will facilitate medical research into psychiatric diseases by providing biological samples along with the associated clinical data, including medical and genealogical information and information on environmental factors, such as descriptors of an individual’s lifestyle.

The computational infrastructure necessary for the bioinformatical analysis of large volumes of genetic information is available at the Computational Center of the Technical Park of SPBU, which has been routinely performing such analyses of the data from the SPBU Biobank since 2015 [78, 79].

## Institute of Translational Biomedicine (SPBU) as a translational core for the RNCPG

The Institute of Translational Biomedicine, SPBU, is currently developing transgenic animal models of neuropsychiatric disorders. It is collaborating closely with SPBU biobank and plans to become a translational core for the Consortium. The modern animal facility provides standard pathogen-free conditions and allows neuropsychiatric disorders to be modeled through targeted mutations in neurotransmitter systems. Several transgenic mouse and rat models are available in the facility, including recently created novel models of enhanced central dopaminergic function based on knockout of the dopamine transporter (DAT) gene in rats [80]. This model has translational relevance to brain disorders classically associated with the enhanced dopaminergic function, such as SZ and attention deficit hyperactivity disorder.

## The RNCPG: getting started

In 2017, the RNCPG started collaborating with the PGC on a GWAS in SZ, and to date it has genotyped 505 SZ patients and 503 population-matched controls with the Infinium Global Screening Array. Next, we plan to perform the first GWAS in a Russian population with SZ.

In 2019, the RNCPG intends to start working with the PGC on a GWAS of alcohol dependence, and in 2020, on major depressive disorder; these will be the first GWASs of these disorders in the Russian population. In 2019, the RNCPG also plans to start collaborating with ConLiGen (www.conligen.org), the International Consortium on Lithium Genetics [81].

## The major planned projects of RNCPG

We are ready to start planning some linked framework projects that are based on modern concepts in both clinical psychiatry and human genomics. The main part of these projects should be prospective and multicenter. We are interested in any kind of international collaborative project and welcome colleagues to join us.

### Deep and precise phenotyping

Another planned project is the deep and precise phenotyping of patients, their families, and high-risk individuals, with a main focus on patients with first-episode SZ. All the available information on multidimensional phenotypes (social and demographic, family and family history, personality, psychometric, clinical, psychiatric and somatic comorbidity, cognitive, laboratory, and neuroimaging information, etc.) will be assessed, and topics of interest will include gene-environment interactions. We will analyze genetic influences on disease course, specific features of disease manifestation, effects and side effects of treatment, remission, and outcome. In 2019, the RNCPG will join forces with the Institute of Psychiatric Phenomics and Genomics (IPPG) at the University Hospital of the LMU Munich (Germany) to perform longitudinal research on recovery-related phenotypes.

### Pharmacogenomics of antipsychotics and antidepressants

In addition, we plan to study the pharmacogenomics of antipsychotics and antidepressants, i.e., “treatment genomics”. This project will be closely linked to the deep and precise phenotyping project and will focus on the genetic control of treatment effects and side effects, with the following key topics: (1) pharmacotherapy, with a special focus on long-acting and depot preparations; (2) transcranial magnetic stimulation (TMS) and electroconvulsive therapy (ECT); (3) psychotherapy alone and in combination with pharmacotherapy.

The first prospective study on treatment-resistant major depressive disorder in the Russian population was started in 2017 as a project within the RNCPG. This naturalistic multicenter study is focusing on mechanisms of treatment resistance rather than on the efficacy of the selected drugs and augmentation therapy. Up to 3000 patients who fulfil ICD-10 diagnostic criteria for a major depressive episode (F32) or major depressive disorder (F33) are to be evaluated. It is planned that the first GWAS in major depressive disorder in the Russian population will be part of this project.

In all of our projects, special attention will be paid to metabolic side effects of psychopharmacotherapy that affect the development and outcome of comorbid somatic diseases in psychiatric patients.

### Psychiatric and somatic comorbidity

In our planned project on psychiatric and somatic comorbidity, we will take a cross-disorder approach. This project will be linked to the “deep and precise phenotyping” and “treatment genomics” projects and focus on the genetic basis of pathogenetic links between common somatic and psychiatric diseases (depression). The specific aim of this project is to use genomics and phenomics data to evaluate whether there is a bidirectional and/or causal relationship between depression and somatic disease.

### Brain biobank

So far, we have only taken the initial steps to start collecting brain samples at the SPBU biobank, and we now plan to increase efforts within the consortium to expand the collection of postmortem brains from psychiatric patients. Currently, we are in the preparatory stage for developing protocols for collecting and storing various postmortem specimens, including brain tissue biopsies. We plan to start collaborations with the Neurobiobank in Munich, Germany (https://www.neuropathologie.med.uni-muenchen.de/neurobiobank_muenchen/index.html) and the Douglas-Bell Canada Brain Bank in Montreal, Canada (www.douglasbrainbank.ca).

## Challenges to address in the RNCPG

The establishment of the RNCPG is the first but important initial step in the long road to achieve our aims and goals. This initiative is the result of our understanding that only joint efforts can enable us to promote and develop psychiatric genetics and genomics in the Russian Federation. We wish to highlight the following are specific challenges facing our Consortium:

1. **Population and geography**. The Russian Federation covers a large geographical area that is located in two continents: Europe and Asia. We need to study psychiatric genetics in heterogeneous populations in historically mixed (European-Asian) regions (Volga-Ural Region, Siberia) that are locally diverse and in more homogenous parts of the country (European region) and thereby pay special attention to the cultural diversity.
2. **Legislation regulation**. To date, the Russian Federation has no specific legislation on the biobanking of human biomaterial, and there are only separate documents regulating the collection, storage, and use of biological samples for certain practical medical purposes (transplantation, reproductive technologies).
3. **Infrastructure**. The further development of our Consortium requires a lot of work to build the next generation of modern scientific infrastructure in the Russian Federation. To address this challenge, we have designed a detailed road map for the initial period up to 2020 on the basis of the available potential of the research centers, St. Petersburg University and the Biobank.
4. **Personnel and educational programs**. The structure of our Consortium needs qualified and experienced personnel in the fields of psychiatry, genetics, bioinformatics, and scientific management. An important goal of the Consortium is to provide adequate training for young mental health care professionals. The main tasks of the unified training programs are to provide recent data on psychiatric genetics and neuropsychiatric research and to exchange study experiences with different centers, regions, and countries. Currently, training is offered as regular annual meetings, conferences, and schools for young scientists and lectures that aim to broaden the understanding of psychiatric genetics and provide education on how to organize clinical trials, develop study protocols and write scientific publications. Most centers already have the resources to provide regular on-site training for young professionals. Trainees are provided with opportunities for integrative learning through visits to different centers and by sharing their experiences. Another important aspect is the constant communication between the trainers. We plan to increase the number of lectures and round tables and will hold annual lectures to provide updates on the work of the Consortium and its current projects and on changes in research methods. In addition, we are considering holding integrated telecommunication conferences to facilitate long-term, ongoing training. One of the important current objectives of the Consortium is to arrange adequate financial support for the continuing education efforts and to involve young specialists in Consortium projects.
5. **Development of international collaborations**. Most of the RNCPG members have a history of successful international collaborations, which provides us with opportunities to develop new collaboration projects worldwide. The Consortium was founded as an open system, and our standards for research design, precision phenotyping, and genetic analysis fully correspond with international ones. All the projects within the RNCPG are flexible enough to enable them to be harmonized with and adjusted to the most modern projects in the field of psychiatric genetics in other countries. We are prepared for any kinds of scientific collaborations and would like to invite all colleagues to participate in joint research programs.
6. **Enlarging the collections of samples and increasing funding**. The RNCPG members are currently funded by government foundation agencies, mostly the Russian Foundation for Basic Research (RFBR) and the Russian Science Foundation (RSF). This funding is for the RNCPG’s own projects within the framework of its goals. Our plans to substantially increase the size of our collections of samples will require increased funding for storage, processing, and data analysis. The successful outcome of the initial steps of the RNCPG (2018–2020) will help us to generate new opportunities for large research projects for the whole Consortium.

## Conclusion

The RNCPG has all the capabilities to achieve its goals. Its members are open to any kinds of collaboration, both within Russia and internationally, that are performed according to the principles of scientific partnership. We believe that high quality genetic, genomic, and pharmacogenetic data from the Russian population are of great value and will be extremely useful for the psychiatric genetics community to allow sustained progress in this fascinating area of human genetics.

## Footnotes

1 We will use these institutions’ abbreviations in the text below.
